# Supplementary material for: Modification Role of Dietary Antioxidants in the Association of High Red Meat Intake and Lung Cancer Risk: Evidence from a Cancer Screening Trial
Source: Antioxidants (Basel). 2024 Jun 30;13(7):799. doi: 10.3390/antiox13070799 (PMC11273743; doi:10.3390/antiox13070799)
Supplement: Supplementary file 1 [file antioxidants-13-00799-s001.zip › antioxidants-3049341-supplementary.pdf]

Figure S1. Flow chart of participants selection [a]

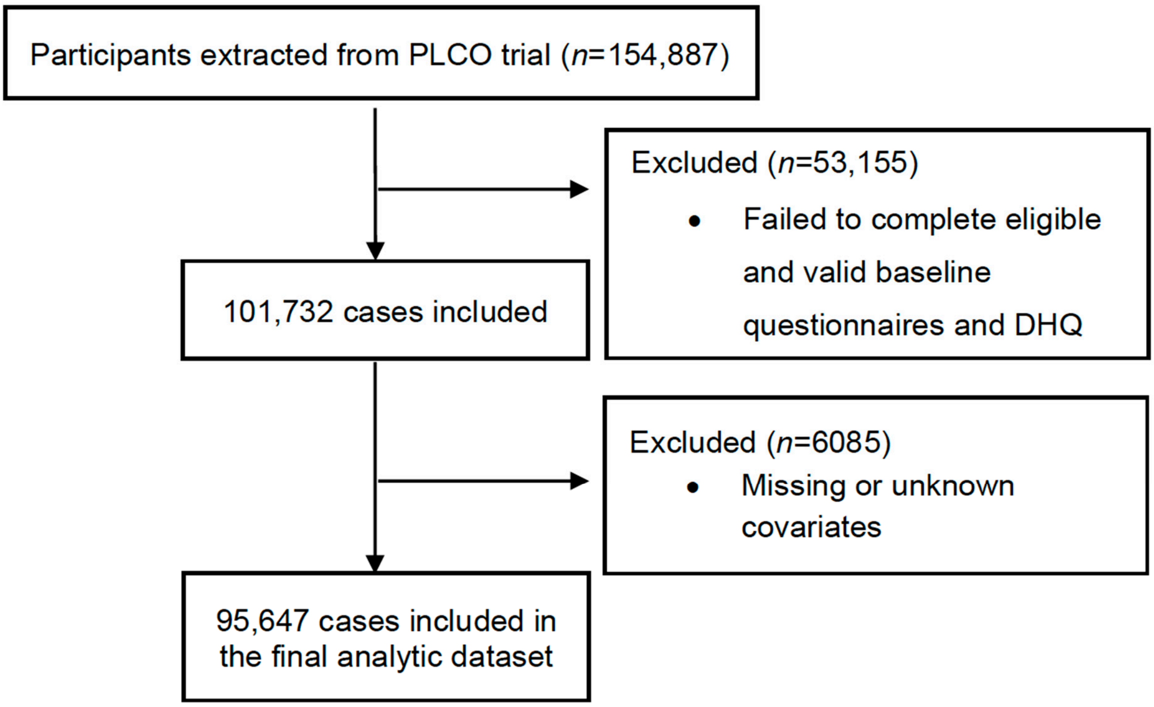

[a] Abbreviations: PLCO, Prostate, Lung, Colorectal, and Ovarian; DHQ, dietary history questionnaire.

Table S1. HRs of lung cancer to red meat intake by quartile, stratified by levels of antioxidant intake from foods, supplements, or both

	Categories of red meat consumption by quartile (g/day)				p-trend
	1 (< 27)	2 (27-48)	3 (48-81)	4 (>81)	
**Intake from diet [a]**					
**fCDAI**					
Low	1 (ref)	1.21 (0.98, 1.49)	1.21 (0.94, 1.55)	1.55 (1.12, 2.14)	0.014
Medium	1 (ref)	1.15 (0.86, 1.54)	1.26 (0.94, 1.68)	1.54 (1.12, 2.12)	0.006
High	1 (ref)	0.94 (0.65, 1.36)	1.10 (0.79, 1.53)	1.24 (0.90, 1.72)	0.080
**Vitamin A**					
Low	1 (ref)	1.37 (1.10, 1.72)	1.44 (1.13, 1.85)	1.76 (1.32, 2.36)	<0.001
Medium	1 (ref)	0.93 (0.70, 1.23)	0.92 (0.69, 1.22)	1.03 (0.75, 1.41)	0.843
High	1 (ref)	1.02 (0.74, 1.40)	1.24 (0.92, 1.68)	1.32 (0.96, 1.84)	0.055
**Vitamin C**					
Low	1 (ref)	1.31 (1.04, 1.65)	1.29 (1.01, 1.65)	1.56 (1.18, 2.07)	0.004
Medium	1 (ref)	1.24 (0.93, 1.67)	1.32 (0.98, 1.78)	1.37 (0.97, 1.94)	0.077
High	1 (ref)	0.86 (0.64, 1.15)	1.01 (0.76, 1.34)	1.12 (0.82, 1.53)	0.362
**Vitamin E**					
Low	1 (ref)	1.22 (0.99, 1.51)	1.29 (1.02, 1.65)	1.62 (1.19, 2.18)	0.002
Medium	1 (ref)	1.16 (0.87, 1.54)	1.11 (0.83, 1.48)	1.39 (1.01, 1.91)	0.063
High	1 (ref)	0.98 (0.68, 1.40)	1.23 (0.89, 1.71)	1.29 (0.92, 1.80)	0.064
**Zinc**					
Low	1 (ref)	1.16 (0.95, 1.42)	1.30 (1.02, 1.67)	2.01 (1.24. 3.27)	0.004
Medium	1 (ref)	1.18 (0.87, 1.61)	1.29 (0.97, 1.73)	1.82 (1.33, 2.48)	<0.001
High	1 (ref)	1.10 (0.73, 1.66)	1.03 (0.71, 1.51)	1.16 (0.81, 1.67)	0.389
**Magnesium**					
Low	1 (ref)	1.22 (0.97, 1.53)	1.37 (1.05, 1.79)	1.26 (0.86, 1.83)	0.054
Medium	1 (ref)	1.28 (0.98, 1.68)	1.15 (0.87, 1.52)	1.64 (1.20, 2.25)	0.010
High	1 (ref)	0.93 (0.66, 1.29)	1.18 (0.88, 1.60)	1.32 (0.98, 1.79)	0.015
**Selenium**					
Low	1 (ref)	1.27 (1.04, 1.55)	1.48 (1.14, 1.91)	1.34 (0.75, 2.38)	0.003
Medium	1 (ref)	0.94 (0.71, 1.25)	0.94 (0.72, 1.23)	1.39 (1.03, 1.86)	0.029
High	1 (ref)	1.12 (0.72, 1.75)	1.30 (0.87, 1.92)	1.43 (0.98, 2.09)	0.027
**Intake from supplements [b]**					
**Vitamin A**					
Low	1 (ref)	1.16 (0.88, 1.52)	1.06 (0.80, 1.41)	1.44 (1.06, 1.96)	0.044
Medium	1 (ref)	1.20 (0.92, 1.56)	1.34 (1.02, 1.76)	1.36 (1.00, 1.86)	0.039
High	1 (ref)	1.05 (0.82, 1.36)	1.23 (0.94, 1.60)	1.35 (0.99, 1.84)	0.037
**Vitamin C**					
Low	1 (ref)	1.14 (0.87, 1.49)	1.14 (0.87, 1.49)	1.31 (0.97, 1.76)	0.103
Medium	1 (ref)	1.04 (0.80, 1.36)	1.15 (0.88, 1.51)	1.32 (0.96, 1.80)	0.074
High	1 (ref)	1.22 (0.94, 1.58)	1.27 (0.96, 1.67)	1.49 (1.09, 2.04)	0.017
**Vitamin E**					
Low	1 (ref)	1.30 (0.99, 1.71)	1.22 (0.92, 1.61)	1.38 (1.02, 1.87)	0.088
Medium	1 (ref)	1.18 (0.90, 1.56)	1.44 (1.09, 1.90)	1.56 (1.14, 2.15)	0.003
High	1 (ref)	0.97 (0.75, 1.25)	1.00 (0.77, 1.31)	1.26 (0.92, 1.72)	0.219
**Zinc**					
Low	1 (ref)	1.02 (0.79, 1.33)	1.01 (0.77, 1.32)	1.25 (0.93, 1.68)	0.177
Medium	1 (ref)	1.17 (0.90, 1.52)	1.33 (0.94, 1.63)	1.28 (0.94, 1.75)	0.116
High	1 (ref)	1.22 (0.93, 1.59)	1.36 (1.04, 1.79)	1.63 (1.19, 2.22)	0.002
**Magnesium**					

Low	1 (ref)	1.08 (0.83, 1.40)	1.10 (0.84, 1.44)	1.31 (0.97, 1.78)	0.091
Medium	1 (ref)	1.28 (0.99, 1.67)	1.15 (0.87, 1.53)	1.40 (1.03, 1.91)	0.082
High	1 (ref)	1.04 (0.80, 1.37)	1.34 (1.02, 1.75)	1.41 (1.04, 1.93)	0.011
**Selenium**					
Low	1 (ref)	1.12 (0.85, 1.46)	1.23 (0.93, 1.63)	1.50 (1.10, 2.04)	0.010
Medium	1 (ref)	1.11 (0.86, 1.45)	1.21 (0.93, 1.58)	1.22 (0.89, 1.66)	0.173
High	1 (ref)	1.22 (0.94, 1.59)	1.18 (0.90, 1.55)	1.50 (1.11, 2.04)	0.019
**Total Intake** [c]					
**Vitamin A**					
Low	1 (ref)	1.39 (1.10, 1.76)	1.28 (0.99, 1.65)	1.60 (1.19, 2.14)	0.007
Medium	1 (ref)	1.10 (0.84, 1.44)	1.19 (0.91, 1.57)	1.47 (1.07, 2.00)	0.014
High	1 (ref)	0.82 (0.60, 1.12)	1.11 (0.83, 1.48)	0.98 (0.70, 1.37)	0.702
**Vitamin C**					
Low	1 (ref)	1.35 (1.05, 1.74)	1.32 (1.01, 1.71)	1.59 (1.18, 2.14)	0.006
Medium	1 (ref)	0.90 (0.67, 1.20)	1.07 (0.80, 1.43)	1.10 (0.79, 1.54)	0.379
High	1 (ref)	1.16 (0.90, 1.50)	1.23 (0.94, 1.61)	1.49 (1.10, 2.03)	0.017
**Vitamin E**					
Low	1 (ref)	1.42 (1.09, 1.84)	1.35 (1.02, 1.77)	1.55 (1.14, 2.10)	0.015
Medium	1 (ref)	1.24 (0.94, 1.64)	1.49 (1.13, 1.98)	1.51 (1.10, 2.09)	0.006
High	1 (ref)	0.91 (0.70, 1.18)	0.97 (0.74, 1.27)	1.31 (0.96, 1.78)	0.137
**Zinc**					
Low	1 (ref)	1.22 (0.97, 1.54)	1.12 (0.87, 1.45)	1.49 (1.11, 2.02)	0.035
Medium	1 (ref)	1.00 (0.77, 1.30)	1.29 (0.98, 1.69)	1.25 (0.89, 1.74)	0.095
High	1 (ref)	1.32 (0.96, 1.82)	1.42 (1.04, 1.93)	1.68 (1.23, 2.30)	0.001
**Magnesium**					
Low	1 (ref)	1.28 (1.01, 1.61)	1.20 (0.92, 1.58)	1.32 (0.93, 1.87)	0.132
Medium	1 (ref)	1.09 (0.83, 1.43)	1.30 (0.99, 1.72)	1.80 (1.32, 2.46)	<0.001
High	1 (ref)	1.09 (0.79, 1.49)	1.22 (0.91, 1.64)	1.31 (0.97, 1.77)	0.061
**Selenium**					
Low	1 (ref)	1.21 (0.99, 1.48)	1.44 (1.13, 1.85)	1.18 (0.68, 2.06)	0.007
Medium	1 (ref)	1.03 (0.77, 1.36)	0.97 (0.73, 1.28)	1.48 (1.10, 1.99)	0.011
High	1 (ref)	1.11 (0.72, 1.71)	1.37 (0.93, 2.01)	1.50 (1.03, 2.17)	0.011

[a] Stratification by diet models adjusted for age, sex, race, study arm, body mass index category, education level, marital status, family history of any cancer, total energy intake, pack-years of smoking, alcohol drinks per day, and supplement use.

[b] Stratification by supplements models adjusted for age, sex, race, study arm, body mass index category, education level, marital status, family history of any cancer, total energy intake, pack-years of smoking, alcohol drinks per day, and fCDAI.

[c] Stratification by total intake models adjusted for age, sex, race, study arm, body mass index category, education level, marital status, family history of any cancer, total energy intake, pack-years of smoking, and alcohol drinks per day.

Figure S2. Spearman correlation of six response variables from reduced rank regression

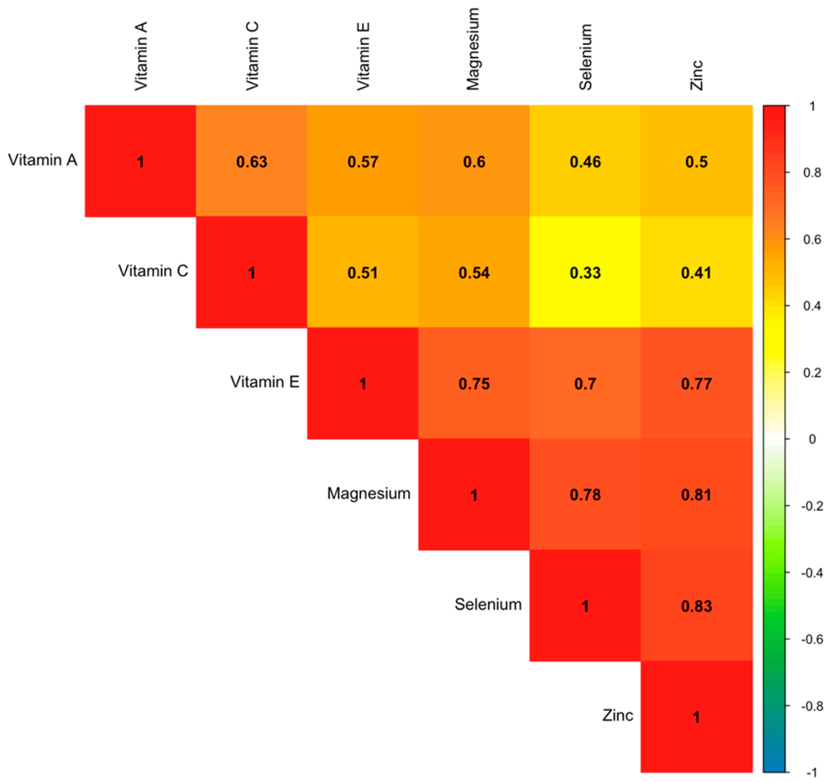

Table S2. Factor loadings between antioxidants intake and consumption of food group (n = 95,647)

Dietary Pattern	
**Food Groups**	**Model Effect Loadings**
Vegetables, total	0.40
Other vegetables	0.14
Non-starchy vegetables	0.36
White potatoes and potato salad	0.21
Fruits, total	0.27
Citrus	0.16
Whole grain	0.19
Pasta	0.21
Cereal	0.15
Beans	0.17
Soy	0.05
Yogurt	0.09
Milk	0.16
Meat, total	0.36
Red meat	0.29
White meat	0.27
Fish, total	0.22
Cream	0.08
Cake	0.16
All sugar/honey	0.04
Sugars and sweets	0.13
Wine	0.03

Table S3. HRs of lung cancer to red meat intake by quartile, stratified by devised dietary pattern score from RRR [a,b,c,d]

	Categories of red meat consumption by quartile (g/day)				P-trend	P - interaction[c]
	1 (<27)	2 (27-48)	3 (48-81)	4 (>81)		
Low	1 (ref)	1.24 (1.01, 1.53)	1.24 (0.97, 1.59)	1.09 (0.82, 1.47)	0.014	0.557
Middle	1 (ref)	1.09 (0.82, 1.47)	1.24 (0.93, 1.65)	1.51 (1.10, 2.08)	0.006	
High	1 (ref)	0.98 (0.68, 1.41)	1.14 (0.82, 1.59)	1.34 (0.97, 1.85)	0.026	

[a] Dietary pattern score devised from RRR were stratified by tertile of the score.

[b] Stratification by dietary score adjusted for age, sex, race, study arm, body mass index category, education level, marital status, family history of any cancer, total energy intake, pack-years of smoking, alcohol drinks per day, and supplement use.

[c] A product term (tertiles of score and quartiles of red meat) was added to the model to examine the statistical evidence of interaction using the likelihood ratio test.

[d] Bonferroni adjusted significance threshold for p-interaction = 0.05/3 (3 levels of antioxidant) = 0.017

Abbreviations: RRR, reduced rank regression
